# Types of cell death and their relations to host immunological pathways

**DOI:** 10.18632/aging.206035

**Published:** 2024-08-08

**Authors:** Kuo-Cheng Lu, Kuo-Wang Tsai, Yu-Kuen Wang, Wan-Chung Hu

**Affiliations:** 1 Department of Medicine, Division of Nephrology, Taipei Tzu Chi Hospital, Buddhist Tzu Chi Medical Foundation, New Taipei City, Taiwan, ROC; 2 Department of Medicine, Division of Nephrology, Fu Jen Catholic University Hospital, School of Medicine, Fu Jen Catholic University, New Taipei City, Taiwan, ROC; 3 Department of Medical Research, Taipei Tzu Chi Hospital, Buddhist Tzu Chi Medical Foundation, New Taipei City 231, Taiwan, ROC; 4 Department of Obstetrics and Gynecology, Taoyuan Armed Forced General Hospital, Taiwan, ROC; 5 Department of Obstetrics and Gynecology, Tri-Service General Hospital, National Defense Medical Center, Taipei, Taiwan, ROC; 6 Department of Clinical pathology, Taipei Tzu Chi Hospital, Buddhist Tzu Chi Medical Foundation, New Taipei City 231, Taiwan, ROC; 7 Department of Biotechnology, Ming Chuan University, Taoyuan City 333, Taiwan, ROC

**Keywords:** apoptosis, autophagy, ferroptosis, necroptosis, NETosis, pyroptosis

## Abstract

Various immune pathways have been identified in the host, including TH1, TH2, TH3, TH9, TH17, TH22, TH1-like, and THαβ immune reactions. While TH2 and TH9 responses primarily target multicellular parasites, host immune pathways directed against viruses, intracellular microorganisms (such as bacteria, protozoa, and fungi), and extracellular microorganisms can employ programmed cell death mechanisms to initiate immune responses or execute effective strategies for pathogen elimination. The types of programmed cell death involved include apoptosis, autophagy, pyroptosis, ferroptosis, necroptosis, and NETosis. Specifically, apoptosis is associated with host anti-virus eradicable THαβ immunity, autophagy with host anti-virus tolerable TH3 immunity, pyroptosis with host anti-intracellular microorganism eradicable TH1 immunity, ferroptosis with host anti-intracellular microorganism tolerable TH1-like immunity, necroptosis with host anti-extracellular microorganism eradicable TH22 immunity, and NETosis with host anti-extracellular microorganism tolerable TH17 immunity.

## INTRODUCTION

Cell death is an essential cellular process that plays crucial roles in development and immune responses. Apoptosis is a well-known example of cell death. Cytotoxic T cells and natural killer cells can utilize the apoptosis mechanism to kill virus-infected host cells. During apoptosis, DNA fragmentation can destroy the viral genome, eliminating viral pathogens. After the discovery of apoptosis, other cell death pathways have been discovered, including autophagy, ferroptosis, pyroptosis, necroptosis, and NETosis [1]. We have proposed a framework encompassing all discovered host immunological pathways, such as TH1, TH2a, TH2b, TH3, TH9, TH17, TH22, TH1-like, and THαβ immune reactions [2–4]. These immune responses combat different types of pathogens and are linked to four types of hypersensitivities. TH1 and TH1-like immune responses fight against intracellular microorganisms, including intracellular bacteria, protozoa, and fungi. TH1 immunity is an eradicable immune reaction, while TH1-like is a tolerable immune reaction. TH1 and TH1-like immune responses are associated with type 4 delayed-type hypersensitivities. TH2 and TH9 immune responses combat parasites, including ectoparasites (insects) and endoparasites (helminths). TH2 immunity is an eradicable immune reaction, and TH9 is a tolerable immune reaction. TH2 and TH9 immune responses are associated with type 1 allergic hypersensitivities. TH22 and TH17 immune responses fight against extracellular microorganisms, including extracellular bacteria, protozoa, and fungi. TH22 immunity is an eradicable immune reaction, and TH17 is a tolerable immune reaction. TH22 and TH17 immune responses are associated with type 3 immune complex-related hypersensitivities. THαβ and TH3 immune responses combat infectious particles, including viruses and prions. THαβ immunity is an eradicable immune reaction, and TH3 is a tolerable immune reaction. THαβ and TH3 immune responses are associated with type 2 antibody-dependent cytotoxic hypersensitivities. Programmed cell death is a crucial component of the host defense mechanism. Thus, different types of host immunological reactions can be related to different types of programmed cell death to defend against different pathogens. Here, we will review these cell death pathways associated with the host immunological pathways.

## Overview of cell death pathways

### Apoptosis

Apoptosis, the earliest discovered cell death pathway, stands in contrast to necrosis, an unprogrammed form of cell death induced by pathogens or various external factors. Unlike necrosis, apoptosis is a tightly regulated and programmed cell death pathway governed by genetic machinery. During embryonic development, apoptosis plays a crucial role as embryonic cells utilize this mechanism to eliminate unwanted cells. Moreover, the apoptosis mechanism is employed in host immune responses to combat pathogenic infections. For instance, the natural killer cell antibody-dependent cellular cytotoxic reaction uses apoptosis to eliminate virus-infected cells. Consequently, apoptosis emerges as a vital component in the body’s self-defense reactions.

Apoptosis can be categorized into two main pathways: the extrinsic pathway and the intrinsic pathway. The extrinsic pathway is activated by external signal molecules, initiating the apoptosis machinery. A classic example of the extrinsic apoptosis pathway involves the interaction between Fas and Fas ligand. On the other hand, the intrinsic pathway is activated by internal cellular signal molecules, with the release of cytochrome c from mitochondria being a characteristic event. Cytosolic cytochrome c serves as a trigger for cell apoptosis. Notably, there is an interconnection between the extrinsic and intrinsic pathways, converging into a common cell death pathway. The apoptosis machinery comprises initiator and executor caspases responsible for breaking down intracellular DNA and proteins. Initiator caspases include caspase 2, 8, 9, and 10, while executor caspases include caspase 3, 6, and 7. This intricate system underscores the convergence of both extrinsic and intrinsic apoptosis pathways into a unified mechanism of cell death.

### Autophagy

Autophagy is a natural, conserved cellular process that digests unwanted, damaged, or old organelles through a lysosome-dependent regulated mechanism. It is referred to as type 2 cell death. Autophagy can be initiated during starvation or other cellular stress situations [5]. It is a process that recycles cell contents to maintain the required metabolism of cells. Special organelles involved in autophagy include mitophagy, the autophagy of mitochondria, and others. Autophagy is an essential cellular process. Autophagic death is a cellular process involving autophagy-induced programmed cell death. This machinery also plays a crucial role in the cell’s defense mechanism [6]. Although initially recognized as a principal degradation pathway to protect against starvation, it is now evident that autophagy also plays a vital role in the homeostasis of non-starved cells. Defects in autophagy are associated with various human diseases, especially neurodegenerative disorders, and modulating autophagy becomes a potential treatment for these detrimental illnesses.

Four types of autophagy have been classified: macroautophagy, microautophagy, chaperone-mediated autophagy (CMA), and crinophagy. In macroautophagy, cytoplasmic components such as mitochondria are targeted and isolated from the main part of the cell within a double-membrane autophagosome. Then, it fuses with a lysosome to become an autolysosome, and eventually, the contents of the vesicle are degraded and recycled. Compared to crinophagy, unnecessary secretory granules are degraded and recycled. In disease, autophagy has been seen as an adaptive response to stress, promoting cell survival; but in other conditions, it could promote cell death and morbidity. In extreme hunger, the breakdown of cellular components promotes cell survival by maintaining cellular energy.

### Pyroptosis

Pyroptosis is another form of programmed cell death [7]. It is related to interleukin-1 and interleukin-18. It is associated with programmed cell death of macrophages. This process can help rapidly clear intracellular pathogens. Pyroptosis usually occurs in immune cells, keratinocytes, and sometimes epithelial cells. This process is triggered by the formation of an inflammasome complex (pyroptosome complex) via the stimulation of intracellular danger signals. The pyroptosome complex is related to the activation of caspases 1/4/5 in humans, which are different caspase sets compared to apoptosis. Caspases 1/4/5 cause the maturation of pro-inflammatory cytokines interleukin-1β and interleukin-18. These caspases also activate the pore-forming protein gasdermin D (GSDMD). Gasdermin D is the key effector molecule of pyroptosis. The inflammasome pathway can be canonical or noncanonical. The canonical pathway involves the activation of pathogen-associated molecular patterns (PAMPs) and damage-associated molecular patterns (DAMPs) recognized by several endogenous pattern recognition receptors (PRRs). For example, NLRP3 or NLRC4 protein is activated by different PAMPs and DAMPs. These receptors can upregulate pro-inflammatory cytokines, including interleukin-12, via the NFkB and MAPK signaling mechanisms. Then, pro-IL-1β and pro-IL-18 are released to be activated via the action of cysteine-regulated caspase-1. Both NLRC4 and procaspase-1 contain a caspase activation and recruitment domain (CARD). After NLRC4 recruits pro-caspase-1, the homotypic CARD-CARD interaction will induce an autocatalytic reaction, allowing pro-caspase-1 to become active caspase-1. Activated caspase-1 cleaves pro-IL-1β and pro-IL-18, enabling these two cytokines to become activated forms. Besides, caspase-1 also cleaves the intracellular gasdermin D. GSDMD will be cleaved into two fragments: the N-terminal GSDMD-N and the C-terminal GSDMD-C. GSDMD-N can form transmembrane pores. These transmembrane pores allow the secretion of IL-1β and IL-18 into extracellular spaces. These pores also impair the extracellular-intracellular ion gradients, causing an increase in osmotic pressure with the influx of water, leading to cell swelling and bursting, resulting in pyroptosis. It is worth noting that GSDMD-N can only insert itself into the inner membrane of specific lipid compositions. And, without cleavage, GSDMD-N is autoinhibited by GSDMD-C. The noncanonical pathway involves the interaction of bacterial lipopolysaccharide and human caspase 4/5. Binding LPS to these caspases induces oligomerization and activation. These caspases also cleave GSDMD to become GSDMD-N, promoting pyroptosis.

### Ferroptosis

Ferroptosis is a type of programmed cell death triggered by excess iron intracellularly. It is characterized by the accumulation of lipid peroxides. Its term is oxytosis. Ferroptosis is triggered by the failure of glutathione-mediated antioxidant defenses. The overall pattern of ferroptosis is the iron-mediated accumulation of oxidatively damaged phospholipids, especially lipid peroxides. When free radicals abstract electrons from a phospholipid, oxidation of phospholipids will occur. Typically, it affects polyunsaturated fatty acids. The main cellular defense mechanism against ferroptosis is mediated by glutathione peroxidase 4 (GPX4). GPX4 can convert lipid peroxides into non-toxic lipid alcohol molecules. Iron is vital and necessary to generate reactive oxygen species to initiate ferroptosis. Thus, treating cells with iron chelators can stop the occurrence of ferroptosis. Additionally, intracellular glutathione (GSH) levels are key to the function of GPX4, so depletion of GSH will lead to ferroptotic cell death. Besides, ferroptosis causes phenotypic changes in mitochondria.

### Necroptosis

Necroptosis is a programmed form of cell death compared to necrosis. The key cytokine mediating necroptosis is TNFα. Binding of TNFα leads to the activation of its receptor TNFR1. TNFR1 receptor binds to TNFR-associated death protein (TRADD) and TNF receptor-associated factor 2 (TRAF2) to activate RIPK1, which recruits RIPK3 to form the necrosome (ripoptosome). During the necroptosis process, the anti-apoptotic protein cFLIP can inactivate caspase 8, facilitating necroptosis. In the absence of caspase 8, RIPK1 and RIPK3 can autophosphorylate and transphosphorylate each other to form a microfilament-like complex named the necrosome. The necrosome phosphorylates the pro-necroptotic protein MLKL, which causes MLKL oligomerization. The oligomerized MLKL will insert into plasma and organelle membranes to induce permeability. Besides, MLKL insertion will induce the leakage of cellular contents of the damage-associated molecular patterns (DAMPs) to trigger inflammation. The necrosome also inhibits the adenine nucleotide translocase in mitochondria, lowering intracellular ATP concentrations. Furthermore, uncoupling of the mitochondrial electron transport chain will lead to mitochondrial damage and open the mitochondrial permeability transition pores, allowing mitochondrial proteins to move into the cytoplasm. The necrosome can additionally cause leaks of lysosomal enzymes into the cytosol via the induction of reactive oxygen radicals by JNK, calpain activation by calcium release, and sphingosine formation. In contrast to apoptosis, the process of necroptosis does not relate to caspase activation. No apoptotic body formation is seen in necroptosis. Cells undergo necroptotic rupture, leaking cellular contents into intercellular spaces.

### NETosis

Neutrophil extracellular traps (NETs) are networks of neutrophil-derived extracellular fibers binding to extracellular pathogens [8]. NETs allow neutrophils to kill extracellular microorganisms with minimal damage to the body [9]. NETs consist of DNA stretches and proteins, including azurophilic granules (neutrophil elastase, cathepsin G, and myeloperoxidase), tertiary granules (gelatinase), and specific granules (lactoferrin). NETs can also form intravascularly via the regulation of platelets. Platelet TLR4 can bind to extracellular microorganisms and activate neutrophils to initiate NETs. Thus, NETs can capture bacteria in blood vessels, stopping their migration via blood circulation. NETs activation and release are usually associated with neutrophil programmed cell death, suicidal NETosis. The NETosis pathway typically begins with NADPH oxidase activation of arginine deiminase 4 (PAD4) via reactive oxygen radicals. PAD4 will induce the citrullination of histones in the neutrophil cell nuclei, resulting in chromatin decondensation. Azurophilic granules (neutrophil elastase, cathepsin G, and myeloperoxidase) enter the neutrophil nucleus and cause the rupture of the nuclear envelope. Then, the decondensed chromatin enters the cytoplasm, where it combines with other cellular granules to form the early-stage NET. NETosis is a double-edged sword, which may cause complications. There is a report suggesting a relationship between NETosis and organ injury [10].

## Overview of host immunological pathways

The immune system is a marvelously complex network, where host immunological pathways play a pivotal role in defending against diverse pathogens. These pathways are categorized based on the dominance of certain immunoglobulins, predominantly into IgG-dominant eradicable immune responses and IgA-dominant tolerable immune responses [2–4, 11]. Eradicable immune responses are initiated by follicular helper T cells (Tfh) via interleukin-21, and tolerable immune responses are initiated by regulatory T cells (Treg) via TGF-β. Understanding the intricacies of these pathways is crucial in comprehending how the immune system combats various threats.

In the realm of eradicable immune responses, the action primarily revolves around combating different types of pathogens through specialized immune mechanisms. The TH1 immunity, for instance, stands guard against intracellular microorganisms such as bacteria, protozoa, and fungi. This branch mobilizes an array of defenders including M1 macrophages, IFNγ-producing CD4 T cells, iNKT1 cells, CD8 T cells (Tc1, EM4), and IgG3 B cells, forming a formidable defense line against these intruders. TH1 immunity is also intricately linked to type 4 delayed type hypersensitivity reactions, highlighting its role in specific immune responses.

In contrast, TH2 immunity gears up against parasites, presenting two distinct subtypes: TH2a and TH2b. TH2a tackles endoparasites (helminths) with its lineup of inflammatory eosinophils (iEOS), interleukin-4/interleukin-5 producing CD4 T cells, mast cells-tryptase (MCt), iNKT2 cells, and IgG4 B cells. On the other hand, TH2b focuses on combating ectoparasites (insects), marshaling basophils, interleukin-13/interleukin-4 producing CD4 T cells, mast cells-tryptase/chymase (MCtc), iNKT2 cells, and IgE B cells. These branches of TH2 immunity are instrumental in addressing parasitic threats and are associated with type 1 allergic hypersensitivity responses.

Expanding further, TH22 immunity is dedicated to countering extracellular microorganisms such as bacteria, protozoa, and fungi. Neutrophils (N1), interleukin-22 producing CD4 T cells, iNKT17 cells, and IgG2 B cells collaboratively orchestrate the defense in this domain. TH22 immunity plays a significant role in type 3 immune complex mediated hypersensitivity reactions, showcasing its specialized function in immune responses.

Moreover, THαβ immunity is specifically tailored to combat infectious particles like viruses and prions [12–15]. This immune pathway employs NK cells (NK1), interleukin-10 producing CD4 T cells, iNKT10 cells, CD8 T cells (Tc2, EM1), and IgG1 B cells to combat these minute yet potent adversaries. Its connection to type 2 antibody-dependent cytotoxic hypersensitivity underscores its significance in addressing infectious threats.

Transitioning to tolerable immune responses dominated by IgA, these pathways exemplify the system’s ability to mount defenses without causing excessive damage to the host. Regulatory T cells play a crucial role in steering these responses, facilitating the switch to IgA, thereby establishing a more tolerable immune milieu.

TH1-like immunity within the tolerable response framework mirrors TH1 immunity but in a more regulated manner. It safeguards against intracellular microorganisms through M2 macrophages, TGFβ/IFNγ-producing CD4 T cells, iNKT1 cells, CD8 T cells (EM3), and IgA1 B cells, while maintaining a balance to prevent hyperactive responses that might harm the host.

TH9 immunity, targeting parasites such as insects and helminths, relies on regulatory eosinophils (rEOS), basophils, interleukin-9 producing CD4 T cells, iNKT2 cells, mast cells (MMC9), and IgA2 B cells to ensure a measured and controlled defense. This pathway, associated with type 1 allergic hypersensitivity, showcases the immune system’s ability to mount responses without tipping the balance toward excessive reactions.

Continuing within the tolerable responses, TH17 immunity is specialized in combating extracellular microorganisms. Neutrophils (N2), interleukin-17 producing CD4 T cells, iNKT17 cells, and IgA2 B cells are the primary players in this pathway, illustrating a fine-tuned defense against extracellular threats while limiting immune-mediated damage through type 3 immune complex mediated hypersensitivity.

Lastly, TH3 immunity within tolerable responses gears up against infectious particles employing NK cells (NK2), interleukin-10/TGFβ-producing CD4 T cells, iNKT10 cells, CD8 T cells (EM2), and IgA1 B cells. This pathway showcases the immune system’s adaptability, mounting responses against infectious particles while maintaining a balanced immune environment to prevent excessive host damage, closely linked to type 2 antibody-dependent cytotoxic hypersensitivity.

**Figure 1:**
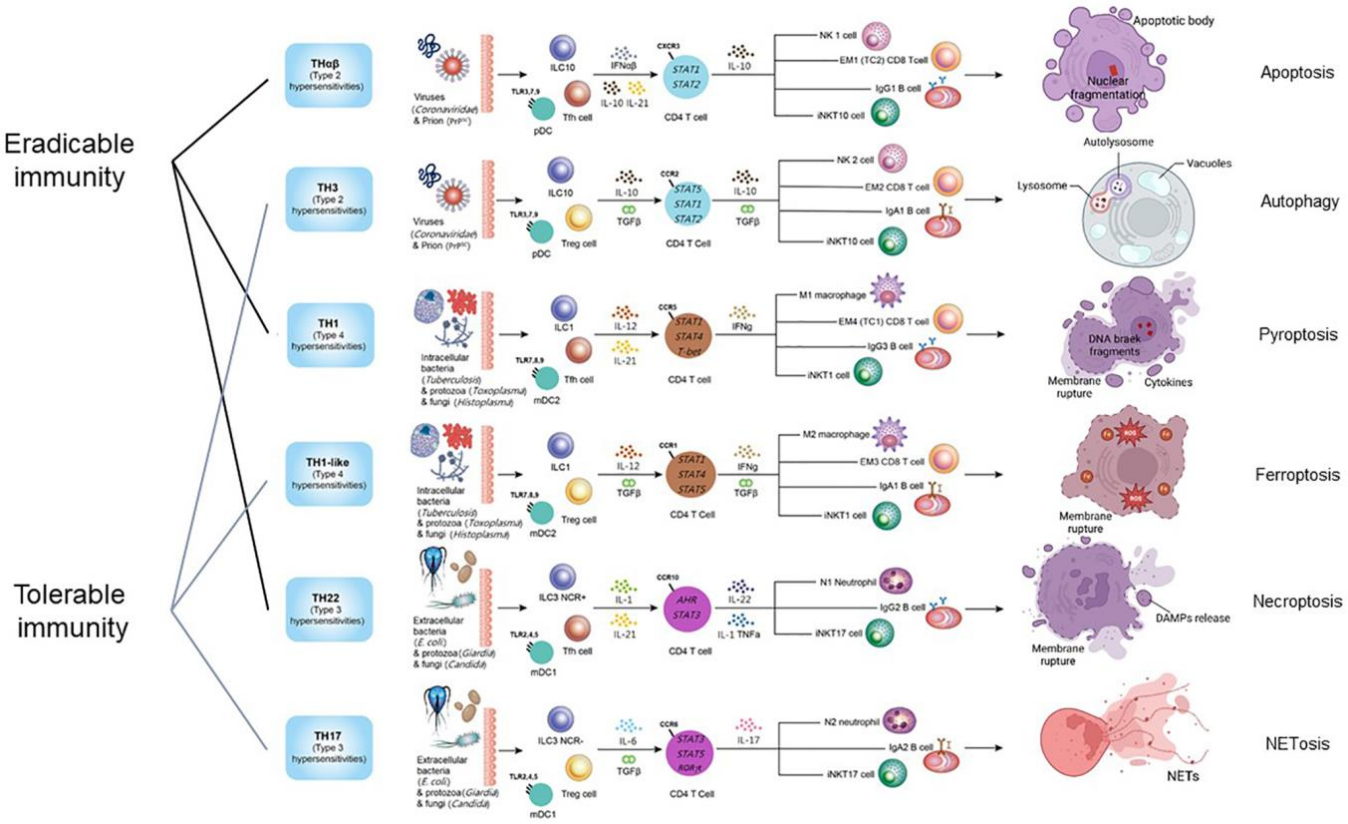
**The framework of host immunological pathways and their relation to programmed cell death.** Apoptosis is related to host anti-virus eradicable THαβ immunity. Autophagy is related to host anti-virus tolerable TH3 immunity. Pyroptosis is related to host anti-intracellular micro-organism eradicable TH1 immunity. Ferroptosis is related to host anti-intracellular micro-organism tolerable TH1-like immunity. Necroptosis is related to host anti-extracellular micro-organism eradicable TH22 immunity. NETosis is related to host anti-extracellular micro-organism tolerable TH17 immunity.

The intricate network of host immunological pathways, categorized into eradicable and tolerable immune responses, showcases the remarkable adaptability and specificity of the immune system in combating diverse pathogens. These pathways not only defend against various threats but also highlight the delicate balance between mounting effective responses and preventing immune-mediated damage to the host. The framework of host immunological pathways and their relations to different types of cell death is shown in Figure 1.

## THαβ immune response and its relation to apoptosis

The host immunological THαβ pathway is the host’s immune reaction against infectious particles, including viruses and prions. Viruses and prions must live intracellularly to replicate and produce more transmissible particles. Apoptosis, the most well-studied programmed cell death pathway, is a key mediator regulating the death of virus-infected cells. During apoptosis, cell death leads to DNA or RNA fragmentation, allowing the intracellular viral DNA or RNA to be destroyed. Thus, virus particles can be eliminated by sacrificing the infected cells. Additionally, activated caspases degrade all intracellular proteins, leading to the destruction of prions, which are protein-based infectious particles, during apoptosis.

THαβ-related immune cells include natural killer (NK) cells, cytotoxic T cells, and IgG1-producing B lymphocytes. NK cells can induce antibody-dependent cellular cytotoxicity (ADCC) of virus-infected cells by binding to IgG1 antibodies [16]. ADCC is an apoptosis mechanism involving DNA and RNA fragmentation. Cytotoxic T cells can also cause apoptosis of virus-infected cells through DNA or RNA fragmentation, thereby killing the viral genomes. This process is mediated by the recognition of viral peptides presented on major histocompatibility complex (MHC) molecules by specific T cell receptors on cytotoxic T cells, which induces the apoptosis machinery. A similar mechanism can be observed in other infectious particles, like prions.

The immunosuppressive cytokine TGFβ has been found to inhibit the apoptosis process [17, 18]. Inhibition of TGFβ signaling can promote NK cell ADCC and cause target cell apoptosis [19]. Conversely, TGFβ can suppress NK cell ADCC. TGFβ-activated kinase 1 (TAK1) can antagonize apoptosis [20]. TGFβ can also inhibit Fas and caspase 8-related apoptosis [21, 22] and induce anti-apoptotic transcription factors to prevent apoptosis. Apoptosis-related protein degradation can lead to the destruction of infectious prion protein pathogens. Additionally, type 1 interferons can induce caspase cascades to trigger apoptosis in malignant cell lines [23–25]. Thus, apoptosis is a THαβ-related host defense mechanism against infectious particles, including viruses and prions.

Furthermore, the THαβ immune response is the host’s eradicable immune reaction induced by follicular helper T cells via the production of interleukin-21. Reports suggest that interleukin-21 is associated with apoptosis, including lymphocyte or cancer cell apoptosis [26–29].

## TH3 immune response and its relation to autophagy

Autophagy is the type 2 programmed cell death pathway and is a milder control mechanism for virus infection of host cells [30–32]. Since type 1 interferons can help control virus infection, research has found a correlation between type 1 interferons and autophagy [24, 33]. Type 1 interferon is an inducer of autophagy [34–36]. Interferon regulatory factor 1 (IRF1), which can activate interferon beta, is also related to autophagy [37].

Autophagy is involved in the presentation of cytosolic antigens to MHC class II molecules and the digestion of intracellularly produced viral protein antigens. Autophagy is a protective mechanism against virus infection by degrading viral particles in autolysosomes. For example, autophagy has been found in liver cells to protect against hepatic virus infection [38]. Hepatitis C virus induces autophagy and interferes with the anti-viral innate eradicable immunity [39–41]. In contrast to apoptosis, autophagy with organelle degradation induces mild host inflammation.

Compared to the THαβ eradicable host immune reaction, the TH3 immunological pathway is the host’s tolerable immune response against viruses and prions. During autophagy, organelles containing virus particles are degraded. Autophagy is often observed in chronic viral infections. The key cytokines in the TH3 immunological pathway are interleukin-10 and TGF-β. However, interleukin-10 is more important for the eradicable THαβ immunity. Research has reported that interleukin-10 can prevent autophagy, and neutralization of interleukin-10 can recover the cellular machinery of autophagy [42–44].

Previous studies have found that TGF-β can promote autophagy [45]. TGF-β can prevent caspase 8-induced apoptosis and induce cell autophagy. TGF-β is mainly produced by regulatory T cells (Treg cells), and impaired Treg activity also impairs autophagy activity [46]. Follicular helper T cells (Tfh cells), which produce interleukin-21, have the opposite function of Treg cells. Previous literature reported that interleukin-21 can suppress autophagy [47].

The TH3 immune response is an IgA-dominant immune reaction, and autophagy has been found to be associated with the pathogenesis of IgA nephropathy [48]. This implies that the TH3 immune response could be related to the autophagy pathway. Interleukin-1, a key cytokine of the TH22/TH17 immunity, increases after the TH3-associated autophagy is blocked. Another THαβ/TH3 cytokine, interleukin-27, can also promote autophagy [49, 50].

## TH1 immune response and its relation to pyroptosis

The TH1 immunological pathway is the host’s eradicable immunity against intracellular microorganisms, including intracellular bacteria, protozoa, and fungi. Pyroptosis, a programmed cell death mechanism, defends against intracellular pathogens [51, 52]. The major effector cells of the TH1 immune reaction are macrophages. Pyroptosis is related to the programmed cell death of macrophages. The key TH1 cytokine, interferon-gamma, is related to the activation of pyroptosis. The inflammasome complex in pyroptosis induces the activation of interleukin-1β and interleukin-18, both of which are pro-inflammatory cytokines against microorganisms. Additionally, interleukin-18 can augment the potency of interferon-gamma, which is the key immune mediator of the TH1 immunological pathway. The activation of interleukin-1β and interleukin-18, triggered by the inflammasome, further induces the production of interferon-gamma.

M1 macrophages are the key effector immune cells of TH1 immunity, and a correlation between M1 macrophage polarization and pyroptosis has been noted in previous studies [53, 54]. Interleukin-23, a vital cytokine in triggering TH1 and TH17 immune reactions, is also associated with macrophage pyroptosis [55]. The activation of the inflammasome also causes the upregulation of NF-κB, the master gene for immune activation signaling. Furthermore, the activation of the inflammasome inactivates interleukin-33, a mediator of the TH2 immunological pathway. Pyroptosis can also trigger pore-induced intracellular traps to capture intracellular bacteria, protozoa, and fungi, leading to their clearance [56]. Caspase-1-induced pyroptosis is an innate immune effector machinery fighting against intracellular microorganisms [52]. The immunosuppressive mediator TGF-β can suppress pyroptosis [57, 58].

Moreover, TH1 immunity is the host’s IgG-dominant eradicable immunity induced by follicular helper T cells via interleukin-21. Previous literature reported that interleukin-21 can cause pyroptosis of certain cells like regulatory T cells (Treg cells) [59]. Additionally, IgG immune complexes can induce macrophage pyroptosis by upregulating the expression of GSDMD [60].

## TH1-like immune response and its relation to ferroptosis

Ferroptosis is a programmed cell death process triggered by intracellular iron overload. Iron is a key chemical element that helps the survival of microorganisms. According to increasing evidence, the occurrence of ferroptosis is always accompanied by inflammation. During the infection of microorganisms, including bacteria, protozoa, or fungi, higher concentrations of iron elements lead to worse infection control by the host. High intracellular iron concentrations help the survival of intracellular microorganisms. To reduce the availability of iron for intracellular microorganisms, iron-triggered cell death can sacrifice the infected cells and eliminate the microorganisms. This is the underlying logical principle of ferroptosis.

Chronic iron overload can drive macrophages to polarize into M2 macrophages, the effector cells of the TH1-like immune reaction [61, 62]. During ferroptosis, iron triggers the accumulation of lipid peroxides, causing membrane peroxidation and damage. Thus, the cell membranes of intracellular bacteria, protozoa, or fungi are damaged, leading to their death. This is why ferroptosis is a mechanism to kill and control intracellular microorganism infections. Glutathione peroxidase 4 (GPX4), which can prevent lipid peroxidation, is a protective mechanism against ferroptosis.

Regulatory T cells (Treg cells) with their key effector cytokine TGF-β can induce a tolerable immune response and tissue fibrosis. TGF-β could enhance ferroptosis via further GPX4 inhibition [63]. Additionally, GPX4 can enhance follicular helper T cells to inhibit ferroptosis [64]. There is a linkage between ferroptosis and fibrosis [65]. Chronic inflammation can be related to ferroptosis-associated tissue destruction and subsequent tissue fibrosis. TGF-β inhibitors can inhibit both ferroptosis and fibrosis [66]. Previous literature suggested an association between ferroptosis and tissue fibrosis, including renal fibrosis, pulmonary fibrosis, and liver cirrhosis [67]. For example, SARS-CoV-2 infection of lung epithelial cells can induce ferroptosis and subsequently lead to pulmonary fibrosis.

The TH1 key cytokine interferon-gamma can enhance ferroptosis in cancer cell lines and epithelial cells [68–70]. TH1-like immunity is an IgA-dominant tolerable immune reaction, and ferroptosis has been found to be related to the pathogenesis of IgA nephropathy [71].

## TH22 immune response and its relation to necroptosis

The TH22 immune response is the host’s eradicable immune reaction against extracellular microorganisms, including extracellular bacteria, protozoa, and fungi. The TH22 immune response is associated with pro-inflammatory cytokines, including TNF-α. TNF-α is the key immune cytokine of the TH22 immune reaction that activates neutrophils to kill extracellular microorganisms. TNF-α is also the major mediator that induces necroptosis. The reason for triggering necroptosis, a programmed cell death pathway, can be to initiate a potent pro-inflammatory immune reaction to kill these invading extracellular microorganisms [72]. Macrophage necroptosis is observed in acute bacterial pneumonia caused by *Serratia marcescens, Staphylococcus aureus, Streptococcus pneumoniae, Listeria monocytogenes*, or uropathogenic *Escherichia coli* (UPEC) [73]. Necroptosis is a key function of neutrophils [74].

Another reason for necroptosis is to destroy potential nutrients from host cells to prevent the growth of extracellular microorganisms. TNF-α activates RIP kinases to form the necrosome. Interferon-gamma, which belongs to the TH1 immune response (different from the TH22/TH17 immunity), can downregulate necroptosis. Type 3 innate lymphoid cells, which can help trigger the TH22/TH17 immunity, are associated with necroptosis [75]. Necroptosis can also stimulate the secretion of TH22/TH17-related pro-inflammatory cytokines. A research study found that TGF-β–activated kinase 1 binding protein 2 (TAB2) deficiency causes dilated cardiomyopathy by enhancing RIPK1-dependent apoptosis and necroptosis [76]. TGF-β is the mediator of tolerable immunological pathways. Thus, eradicable immune mechanisms like apoptosis or necroptosis can be enhanced without TGF-β signaling. Another study pointed out that TGF-β-activated kinase 1 (TAK1) serves as a key survival factor in cardiac organs by directly antagonizing necroptosis [20].

The TH22 immune response is the host’s IgG-mediated eradicable immunity, which is initiated by follicular helper T cells via interleukin-21. There is no direct evidence suggesting that interleukin-21 can induce necroptosis. However, previous literature reported that interleukin-21 can cooperate with TNF-α, the key factor of necroptosis, to induce autoimmune disorders [77]. Additionally, IgG immune complexes have been found to trigger necroptosis in a previous study [78]. Interleukin-22 is the central cytokine of the TH22 immune reaction, and previous research pointed out that interleukin-22-producing type 3 innate lymphoid cells are related to necroptosis [75].

## TH17 immune response and its relation to NETosis

The TH17 host immune reaction is the tolerable immune response against extracellular bacteria, fungi, or protozoa. Neutrophils play dominant roles in the TH17 tolerable immune response. In this situation, neutrophils cannot successfully kill and eradicate these extracellular microorganisms. Thus, these neutrophils sacrifice themselves to stop the progression of these extracellular microorganisms. These polymorphonuclear neutrophils (PMNs) have condensed DNA contents in their cell nuclei, and they trigger the NETosis cell programmed death pathway. Then, these extracellular microorganisms can be entrapped in the neutrophil extracellular traps (NETs), and other alive neutrophils will digest these extracellular bacteria, protozoa, or fungi. NETs can also induce TH17 immune cells [79].

The tolerable antibody IgA is found to activate NETosis. NETosis is also found to be correlated with chronic inflammation and delayed wound healing [80]. The TH17 immune reaction-related IgA immune complex formation is also associated with NETosis via the activation of the Fc-α receptor [81, 82]. IgA vasculitis has also been reported to be associated with NETs [83]. The central cytokine of the TH17 immune response, interleukin-17, can also induce NETosis [84]. Neutrophils can release IL-17 through extracellular trap formation during psoriasis [85]. IL-17A is expressed on NETs in ankylosing spondylitis [86]. The TH22/TH17 key cytokine TNF signaling can induce NETosis of CCR5+ neutrophils [87]. Thus, the TH17 host tolerable immunological pathway is associated with NETosis.

TAK1 is also required for neutrophil extracellular trap formation [88], pointing out the significance of TGF-β in NETosis. TGF-β itself can also induce NETs [89]. Regulatory T cells, characterized by the secretion of TGF-β, suggest that Treg cells are mediators of NETosis. NETs can also directly trigger epithelial and endothelial cell death [90].

## CONCLUSIONS

Programmed cell death pathways are related to different host immunological pathways. Apoptosis is related to the host’s anti-viral eradicable THαβ immunity. Autophagy is related to the host’s anti-viral tolerable TH3 immunity. Pyroptosis is related to the host’s anti-intracellular microorganism eradicable TH1 immunity. Ferroptosis is related to the host’s anti-intracellular microorganism tolerable TH1-like immunity. Necroptosis is related to the host’s anti-extracellular microorganism eradicable TH22 immunity. NETosis is related to the host’s anti-extracellular microorganism tolerable TH17 immunity. These relationships can help us understand the host defense mechanisms against invading pathogens and provide new insights for developing better therapeutic strategies against infections or autoimmune disorders.

### Data availability statement

This review article was performed via literature search without conducting experiments. Data sharing is not applicable to this article as no new data were created or analyzed in this study.
